# Improving the Mechanical Properties of Sulfoaluminate Cement-Based Grouting Material by Incorporating Limestone Powder for a Double Fluid System

**DOI:** 10.3390/ma13214854

**Published:** 2020-10-29

**Authors:** Yanfeng Wang, Songhui Liu, Dongxing Xuan, Xuemao Guan, Haibo Zhang

**Affiliations:** 1 Henan Key Laboratory of Materials on Deep-Earth Engineering, School of Materials Science and Engineering, Henan Polytechnic University, Jiaozuo 454000, China; 418290361@hpu.edu.cn (Y.W.); guanxuemao@hpu.edu.cn (X.G.); 2 Department of Civil and Environmental Engineering, The Hong Kong Polytechnic University, Hung Hom, Kowloon, Hong Kong, China; d.x.xuan@polyu.edu.hk

**Keywords:** sulfoaluminate cement, grouting material, limestone powder, mechanical properties, microstructure

## Abstract

To improve the hardening performance of sulfoaluminate cement-based grouting material (SCGM) and reduce its cost, limestone powder was adopted to replace anhydrite in the control SCGM. The influence of the replacement rate of limestone powder on the hydration, hardening strength, expansion, and microstructure evolution of the SCGM was systematically researched. The results indicated that replacing anhydrite with limestone powder in SCGM can improve the flowability, shorten the setting time, and enhance the compressive strength at early and late stages. When the replacement rate of limestone powder was 20%, the compressive strength of SCGM for 6 h and 28 days increased by 146.41% and 22.33%, respectively. These enhancements were attributed to the fact that fine limestone powder can accelerate the early hydration reaction rate and promote the formation of ettringite due to its nucleation effect. Moreover, due to the presence of limestone powder, mono-carbonate (Mc) can be formed, which would densify the microstructure and refine the pore structure of the hardened SCGM.

## 1. Introduction

Grouting materials are widely used to reinforce and consolidate the broken rocks or soft soil or cavity beneath pavement in underground engineering [1,2,3]. In order to ensure the flowability and permeability of grouting materials into the microcracks, pure slurry without any aggregates is usually used [4]. Common grouting materials include cementitious, resinous, or solution chemical mixtures [2,5,6]. Among them, resinous and solution chemical mixtures are increasingly limited due to their high cost and increasing environmental concerns. As an alternative, cement-based grouting materials are gaining worldwide attention [7,8,9].

Portland cement is commonly used into a cement‑based grouting material due to its worldwide availability and low cost [10]. However, the poor early strength and long setting time of portland cement, especially at a high water to cement ratio, limits its wide application. In recent years, more and more works have focused on the use of sulfoaluminate cement (CSA) to prepare cement-based grouting materials. This is because the sulphoaluminate cement has the advantages of short setting time, high early strength, and micro-expansion [11,12,13]. Commonly, a high water to cement ratio, ranging from 0.5 to 1.5, is used to reduce the cost as aggregates are not used [14]. It is obvious that with the increase of the water to cement ratio, the cost of grouting materials can be significantly decreased and the flowability can be improved. However, a problem of a long setting time and a relativly low early strength exist in practice.

In order to further improve the early hardening performance of sulfoaluminate cement‑based grouting materials, several strategies have been adopted in authors’ previous works, such as double fluid grouting process [3,4], ultra-fine processing of raw materials [15], and adding nano‑nucleating powder [16,17,18]. The double‑fluid grouting process includes preparing slurry A and slurry B, separately. Slurry A mainly includes CSA clinker, retarder and water, while slurry B is a mixture of anhydrite, lime and water. The single slurry A or B with high flowability can last for 20–40 h without setting, but the blended slurry would harden and gain excellent strength quickly after mixing [4]. This technology has been put into practical applications in China. Ultra‑fine processing of raw materials has been proved to be a useful technology to improve the performance of SCGM. After ultra-fine processing of raw materials, the setting time can be shortened, and early hardening strength can be enhanced significantly [3]. Moreover, the reduced particle size and rapid hardening behavior would also help the penetration of the SCGM into microcracks (below 30 μm) and prevent the slurry from leakage. Adding nano nucleating powders, such as LiAl‑layered double hydroxides [17,18], superfine ettringite [1], can also accelerate the hydration reaction and improve the early hardening performance of SCGM due to its nucleation effect. However, the addition of nano-nucleating powder will reduce the flowability of slurry due to its nano size effect. Besides, technical concerns are needed for the dispersion of nano-nucleating powder in SCGM and the cost of the nano‑nucleating powder. Nevertheless, this provides new insights for improving the performance of SCGM by adding nucleating agents.

Limestone powder is commonly used in ordinary Portland cement-based materials as a mineral admixture [19,20]. It has been widely reported that limestone powder could accelerate the hydration of Portland cement due to its nucleating and dilution effects. Moreover, the limestone powder can react with the aluminate phase from cement to form supplementary mono-sulfate (AFm) phases and stabilize ettringite [21,22,23]. The total porosity of the hardened cement pastes can be also reduced, which is attributed to the physical filler effect and increase of the total volume of the hydration phase due to the reaction between limestone powder and cement [24,25]. SCGM contains lots of aluminate phase. Therefore, it could be possible to improve the performance of SCGM by using fine limestone powder. However, there has been little research on the effects of fine limestone powder on the hydration, hardening performance, expansion, and microstructure evolution of the SCGM, especially in the double fluid system with a water to solid ratio greater than 1.

This study aims to investigate the influence of limestone powder to replace anhydrite in SCGM on the hardening performance of SCGM. The tested properties include flowability, setting time, hydration and hardening performance, and volume stability of SCGM. The phase assemblage, porosity, and microstructure evolution were also determined as well to unravel the mechanism in this work.

## 2. Materials and Experimental

### 2.1. Raw Materials

**Figure 1:**
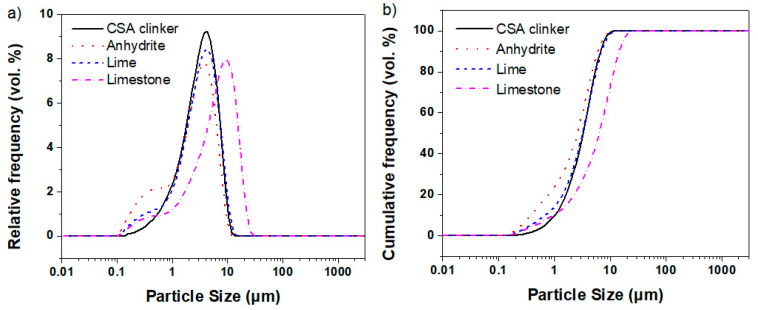
Particle size distribution of raw materials, (**a**) frequency distribution and (**b**) cumulative distribution.

CSA clinker, lime, anhydrite and limestone were used as raw materials to prepare the SCGM. All four raw materials were purchased from a local commercial building materials factory in China (Jiaozuo Dinghao Technology Co., Ltd., Jiaozuo, China). The chemical composition of these four raw materials was determined by X-ray fluorescence (XRF) and is listed in Table 1. The primary minerals of CSA clinker were C_4_A_3_$\bar{S}$, C_2_S and C_4_AF, and the detailed mineral composition is shown in Table 2. The effective amount of calcium sulfate in anhydrite was 85.19 wt.%, and the calcium oxide amount in lime was 64.72 wt.%. The main mineral of limestone was calcite with a purity of 97.23%, as determined by X-ray diffraction analysis (XRD) and Thermal gravimetric analysis (TGA). To improve the early hydration hardening performance and the permeability of SCGM, the raw materials were also ground by using a fluidized bed-type jet mill to achieve a maximum particle size below 30 μm [3]. The particle size distribution of raw materials is shown in Figure 1, as determined by using a Malvern laser sizer 3000 (Mastersizer 3000, Malvern, UK).

### 2.2. Mixture Proportion of SCGM

**Figure 2:**
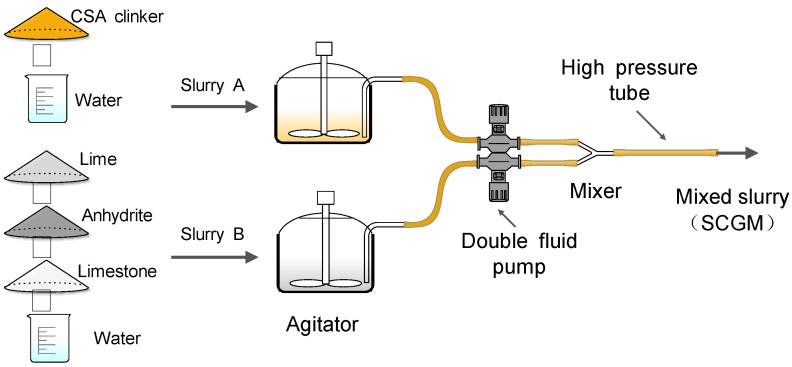
Schematic diagram of sulfoaluminate cement‑based grouting materials for double fluid system.

SCGM included the preparation of slurry A and slurry B separately, as illustrated in Figure 2. Slurry A mainly included CSA clinker and water, while slurry B was a mixture of anhydrite, lime, limestone and water. The detailed mixture proportion of different mix is listed in Table 3. 

The anhydrite in slurry B was replaced with limestone by 0%, 10%, 20%, 30%, and 40%, labeled as L‑00, L‑10, L‑20, L‑30, and L‑40, respectively. The water to solid ratio for slurry A and B were 1.2, and slurry A and B were mixed separately for 3 min. Then the flowability of slurry A and B were tested. After testing flowability, slurry A and slurry B were further mixed for another 1 min at a weight ratio of 1:1. The homogeneously mixed slurry was cast into steel mold with a dimension of 40 mm × 40 mm × 40 mm and 25 mm × 25 mm × 280 mm for compressive strength and expansion-shrinkage test, respectively. After casting, the surface of the slurry samples was covered with a thin plastic wrapper to avoid evaporation of water. After two hours, the prepared samples were de‑molded and covered with plastic wrapper until the required testing age at a constant temperature of 25 °C.

### 2.3. Test Methods

#### 2.3.1. Flowability and Setting Time Test

The flowability of slurry A and slurry B were tested separately by using the Marsh cone method according to our previous work [3]. The flow time of each slurry was recorded. After testing flowability, the slurry A and slurry B were mixed at a weight ratio of 1:1, and the setting time of the mixed slurry was measured by Vicat needle test [3]

#### 2.3.2. Hydration Heat Test

The hydration heat release of each mixture was measured using an isothermal conduction calorimeter (ICC, TAM Air, Newcastle, DE, USA). According to the mixture proportion shown in Table 3, about 4 g of the mixed slurry were poured into a glass ampoule and placed into the calorimeter to record the heat flow and cumulative heat release at a constant temperature of 20 °C for 24 h.

#### 2.3.3. The Compressive Strength of Hardened SCGM

The compressive strength of hardened SCGM with different mixture proportions was tested at the ages of 6 h, 1 day, 3 days, 7 days and 28 days, respectively. For the compressive strength test, a multi‑function mechanical testing machine (Testo-metric CXM 200 kN, Testometric, London, UK) on the cubic samples with a loading rate of 0.6 MPa/s was used [3,16]. Six samples were tested for each mixture proportion, and the average values and standard deviations of the compressive strengths for each mixture proportion were determined.

#### 2.3.4. Expansion and Shrinkage Test

The prismatic samples (25 mm × 25 mm × 280 mm) were used to test the expansion and shrinkage properties of SCGM to evaluate its dimensional stability. After curing for 2 h, these samples were removed from the mold, and the initial length was measured. Then the length changes during the curing process were recorded by using a comparator [3]. The expansion rate was calculated by applying the following equation: $\Delta L\left(\%\right)=\frac{L_{t}-L_{0}}{L_{0}}\times 100$, being *L_t_* the measured length at a given time and *L*_0_ the initial length [26].

#### 2.3.5. Microstructure Analyses

##### X‑ray Diffractometry (XRD)

After compressive strength tests, the crushed particles from the specimens were collected. The collected particles were soaked in isopropanol for 24 h, then dried and stored in a desiccator with silica gel and under vacuum for another 7 days. For the preparation of powder samples, the dried particle samples were ground into a powder with the particle size below 75 μm by using a mortar and pestle. The XRD patterns of the powder samples were recorded by using high-resolution powdered X-ray diffractometer (XRD, Smart Lab, Rigaku, Tokyo, Japan) with Cu-Ka radiation (λ = 1.54 Å) at a range of 5–40 °C with a scan speed of 5°/min at 45 kV and 200 mA.

##### Thermogravimetric Analysis (TGA)

The powder samples used in the XRD test were also used to conduct the TGA test, and approximately 10 mg of powder samples were heated from 40 to 1000 °C at a ramping rate of 10 °C/min with an N_2_ striping gas using a commercially available instrument (Thermo plus EVO2, Rigaku, Tokyo, Japan).

##### Scanning Electron Microscopy (SEM)

The well‑dried particle samples without grinding were used to observe the morphologies of the hardened SCGM by using a scanning electron microscopy (SEM, Merlin Compact, Carl Zeiss NTS GmbH, Oberkohan, Germany) at 20 kV coupled with energy-dispersive X‑ray spectroscopy (EDS, OXFORD, Oxford, UK). Before the SEM observation, the samples were sputter‑coated with a conductive gold layer.

##### Mercury Intrusion Porosimetry (MIP)

The porosity evolution of the hardened SCGM was evaluated by using a mercury intrusion porosimeter (AutoPore IV 9500, Micromeritics, Atlanta, Georgia, USA). The well‑dried particles with a size of 3.35–5.00 mm were used to conduct the MIP test. The porosity and pore size distribution of the samples were obtained by assuming the pores were cylindrical, and the contact angle between mercury and samples was 140°.

## 3. Results and Discussion

### 3.1. Fresh Properties of SCGM

#### 3.1.1. Flowability of Slurry A and Slurry B

**Figure 3:**
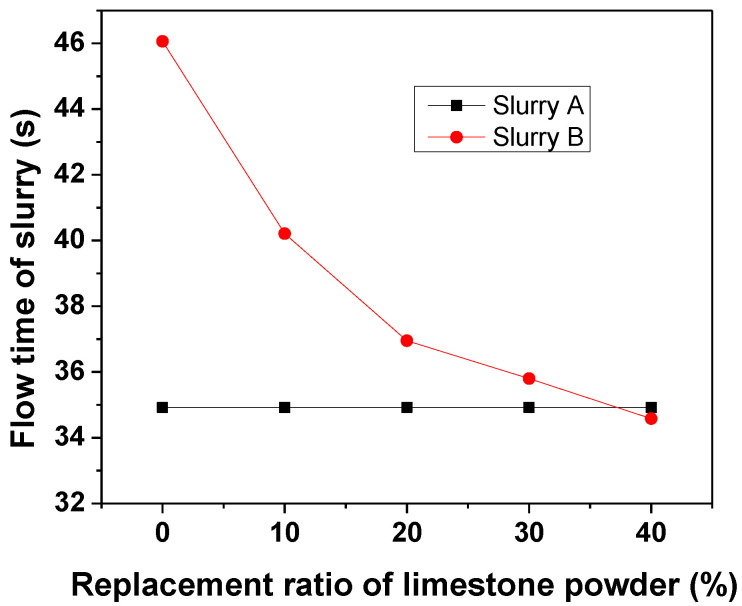
Flow time of slurry B as a function of replacement rate of limestone powder.

Figure 3 shows the Marsh cone flow time of slurry A and slurry B. It was noticed that the flow time of slurry A was 34.9 s, suggesting its excellent flowability. The flow time of the slurry B without limestone replacement was 46.06 s, indicating that the flowability of slurry B was slightly lower than that of slurry A. When the replacement rate of limestone powder increased from 0% to 40%, the flowability of slurry B was greatly improved. Compared with L‑00, the flow time of L‑10, L‑20, L‑30 and L‑40 were reduced by 12.70%, 19.78%, 22.28% and 24.92%, respectively. The improved flowability of the slurry was attributed to two reasons. First, the anhydrite would react with water to form gypsum during the mixing process, in which the water was consumed, as shown in Equation (1). In contrast, the limestone powder was difficult to be dissolved in water. Secondly, the particle size of limestone powder was larger than that of anhydrite, as shown in Figure 1. This means that the water demand of the limestone powder due to surface area was less. As a consequence, when limestone powder was used to replace anhydrite partially, the flowability was significantly improved. Moreover, after replacement, the flowability of slurry B became similar to that of slurry A, which is beneficial to the construction of the designed grouting material.

(1) $${\mathrm{CaSO}}_{4}\;\left(\mathrm{anhydrite}\right)+2H_{2}O\;\to {\mathrm{CaSO}}_{4}\cdot 2H_{2}O\;\left(\mathrm{gypsum}\right)$$

#### 3.1.2. Setting Time

The setting time of the mixed slurry is tabulated in Table 4. The initial setting time and final setting time of the L‑00 sample were 5.0 and 7.5 min, indicating the rapid hardening behavior of SCGM, even at a high water to solid ratio of 1.2. This excellent early hardening behavior was attributed to alkalinity increase of the mixed slurry when an appropriate content of lime was used in slurry B [27] and the ultra‑fine process of raw materials [3]. Interestingly, the setting time of SCGM could be further reduced by partially replacing anhydrite with limestone. It was observed that, for the samples of L‑10, L-20, L-30 and L-40, the initial setting time was reduced by 10%, 46%, 50% and 72%, and the final setting time was reduced by 20%, 26.7%, 33.3% and 36%, respectively. As the replacement rate of limestone powder was increased, the setting time of SCGM was decreased, suggesting that the limestone powder may accelerate the hydration of SCGM due to its nucleating effect [19,24,28].

#### 3.1.3. Heat of Hydration

**Figure 4:**
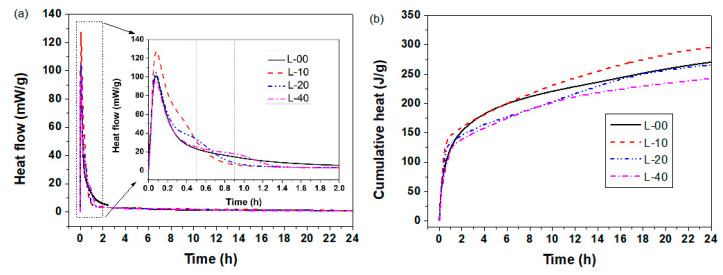
Hydration heat curves of SCGM. (**a**) Heat flow; (**b**) Cumulative heat release.

To revel the effects of limestone powder replacement on the hydration process of SCGM, the hydration heat evolution of L-00, L-10, L-20 and L-40 samples are shown in Figure 4. It was observed that there was only one peak for the L-00 sample, and the heat flow peak was 100.98 mW/g, indicating the hydration of SCGM to form ettringite very fast with heat release [29]. As the replacement rate of limestone powder was increased, the first heat flow peak value was increased first and then decreased. More specifically, the heat flow peak value for the sample of L-10, L-20 and L-40 was 127.60 mW/g, 104.48 mW/g, and 96.01 mW/g, respectively. This means that limestone powder could accelerate the hydration of SCGM [25,29]. When the replacement rate of limestone powder was less than 20%, more ettringite would be formed. However, when the replacement rate of limestone powder exceeded 20%, the formation of ettringite was suppressed due to the reduced anhydrite amount in the mixed slurry, as shown in Equation (2). The cumulative heat curves showed the same trend. Replacing anhydrite with less than 20% limestone would increase the total heat release, but further increasing the replacement rate to 40% would cause a reduced heat release due to the reduced formation of ettringite. The cumulative hydration heat of the L-00, L-10, L-20, and L-40 during the first day was 269 J/g, 296 J/g, 264 J/g, and 241 J/g, respectively. Moreover, in addition to the acceleration effect of limestone powder, it should also be noted that a weak shoulder peak appeared in the sample of L-10, L-20, and L-40, indicating limestone may also take part in the hydration reaction in addition to the physical nucleating effect [27]. Martin [29] found that in the ternary systems containing CSA clinker, anhydrite and limestone, the limestone would participate in the hydration reaction and also accelerate the hydration reaction.

(2) $$C_{4}A_{3}\bar{S}+2C\bar{S}\;\left(\mathrm{anhydrite}\right)+38H\;\to C_{3}A\cdot 3C\bar{S}\cdot H_{32}\;\left(\mathrm{ettringite}\right)+2{\mathrm{AH}}_{3}$$

### 3.2. The Compressive Strength Development

**Figure 5:**
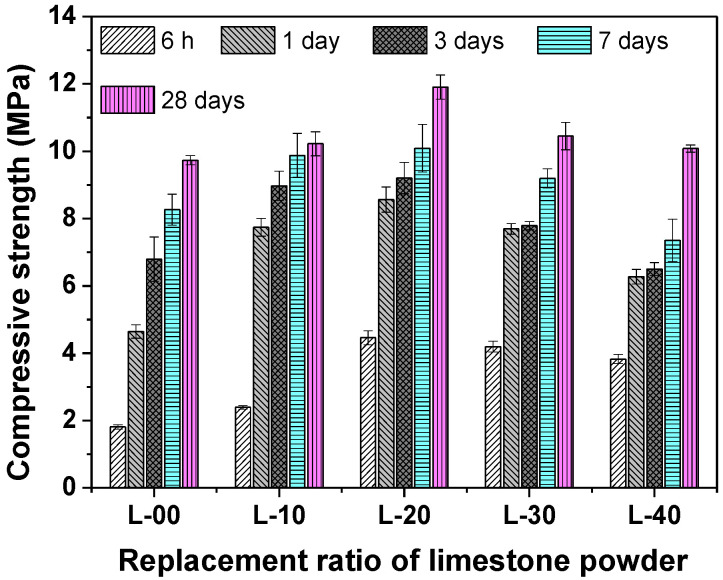
The compressive strength development of SCGM.

Figure 5 shows the compressive strength development of SCGM with different mixture proportions. For the sample of L-00 without limestone powder, the compressive strength for 6 h, 1 day, 3 days, 7 days, and 28 days was 1.81 MPa, 4.65 MPa, 6.79 MPa, 8.26 MPa, and 9.73 MPa, respectively. When replacing anhydrite with less than 20% limestone, both compressive strengths at early and later ages were increased significantly compared with that of the L-00 sample. 

Further increasing the replacement rate to 40%, the later compressive strength was decreased, while the early compressive strength was still increased. Compared with L-00, the compressive strength for 6 h, 1 day, 3 days, 7 days, and 28 days were increased by 146.41%, 84.29%, 35.54%, 22.11%, and 22.33%, respectively. The enhanced early compressive strength was mainly attributed to the accelerated hydration of SCGM, as proved by the setting time and hydration heat results. Moreover, the physical filler effect of limestone powder may also contribute to the compressive strength development of SCGM [27].

### 3.3. Expansion Behavior of SCGM

**Figure 6:**
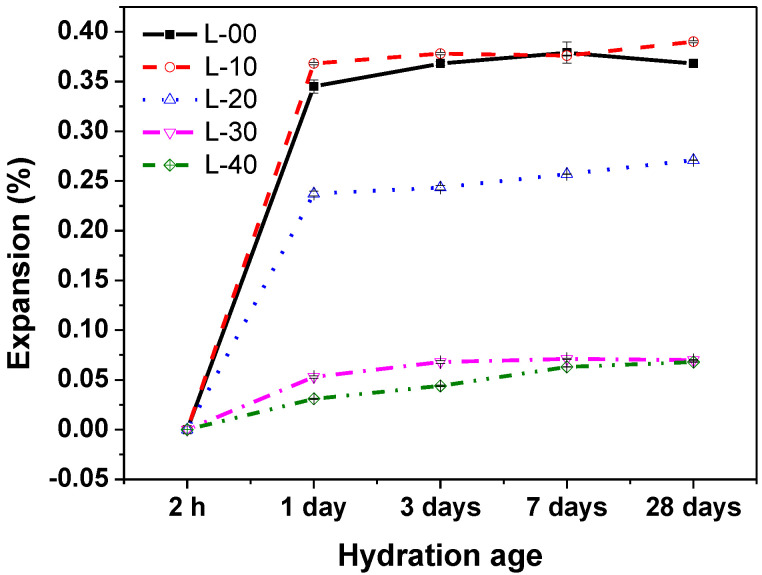
Linear expansion rate of SCGM at different ages.

Figure 6 shows the effects of limestone powder replacements on the expansion behavior of SCGM. All samples showed an expansion as the curing age increased, but the expansion rate reached a plateau after the one‑day curing. When a high water to solid ratio of 1.2 was used in this work, it helped lead to a high hydration degree of SCGM in the early ages [27]. In detail, L-00 samples showed an expansion level of 0.35% by 1 day, and the expansion ratio of L-10 was similar to that of L-00, however, with the replacement rate of limestone powder was increased, the expansion rate was decreased, which is consistent with a previous research in which the expansion in CSA cements were decreased with decreasing calcium sulfate contests [30]. Hargis and Xu [27,31] also found that the expansion of calcium sulfoaluminate-based cementitious systems was reduced by calcite and vaterite, irrespective of the presence of gypsum.

### 3.4. Microstructure Analysis

#### 3.4.1. Phase Assemblage of Hardened SCGM

**Figure 7:**
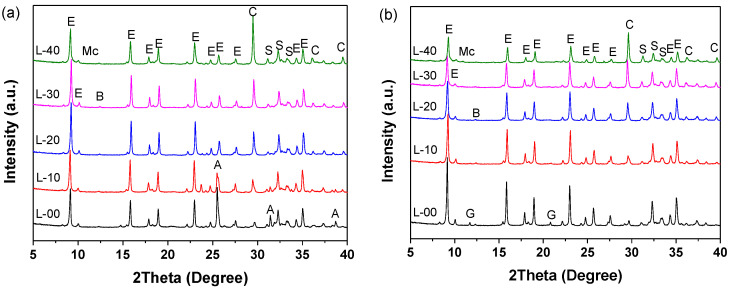
XRD patterns of SCGM after different curing age; (**a**) 6 h; (**b**) 28 days. (E: Ettringite; Mc: Mono-carbonate; C: Calcite; A: Anhydrite; G: Gypsum; S: C_2_S; B: C_4_AF).

**Figure 8:**
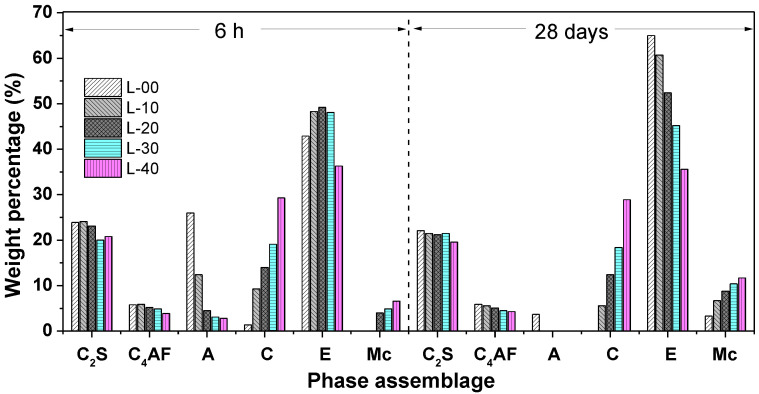
Rietveld quantitative analysis of the crystalline phase in SCGM after 6 h and 28 days. (A: Anhydrite; G: Gypsum; C: Calcite; E: Ettringite; Mc: Monocarboaluminate). Note that the amorphous phase (mainly Al(OH)_3_ gel) is not quantified.

Figure 7 shows the XRD patterns of SCGM after curing for 6 h and 28 days, and the crystalline phases by QXRD are shown in Figure 8. From Figure 7, it was observed that there was no diffraction of C_4_A_3_$\bar{S}$ existed in all samples, and strong diffraction peaks of ettringite appeared even after hydration curing for 6 h. This indicated that the C_4_A_3_$\bar{S}$ mineral in CSA clinker could react with anhydrite to form ettringite rapidly at a high water to solid ratio, as shown in Equation (2) [26]. For the samples cured for 6 h, it was also observed that with increasing the replacement rate of limestone powder, the intensity of ettringite increased first and then decreased. The increased rate of ettringite for the sample of L‑10, L‑20, L‑30 and L‑40 was 12.59%, 14.69%, 12.12%, and −15.38%, respectively, compared with the L-00 sample, as shown in Figure 8. This means that the limestone powder would promote the formation of ettringite in the early ages [25,27,29,31], which explains why the limestone powder would accelerate the hydration of SCGM and contribute to the early strength development. However, for 28‑day curing, the intensity of ettringite was decreased with the increasing replacement rate of limestone powder due to the dilution effect of limestone powder and the reduced anhydrite amount in SCGM. The reduced rate of ettringite for the sample of L‑10, L‑20, L‑30 and L‑40 was 6.62%, 19.38%, 30.46%, and 45.23%, respectively, compared with the L‑00 sample, as shown in Figure 8.

Moreover, it should be noted that with the increase in the replacement rate of limestone powder, a weak peak of mono-carbonate (Mc) appeared in the samples of L‑10, L‑20, L‑30, and L‑40 for 6 h and 28 days, and the amount of calcite was consumed, as shown in Figure 8. This could be related to the reaction between calcite and C_4_A_3_$\bar{S}$ mineral to form Mc, as shown in Equation (3) [25]. It was widely reported that the presence of limestone powder led to the formation of mono-carbonate, which stabilized the ettringite and increased the total volume of hydrates products [20,21,24,32]. There were also lots of unreacted anhydrite in the L‑00 sample cured for 6 h, which turned to gypsum after further curing for 28 days. From Figure 8, it was also observed that the weight percentage of C_2_S and C_4_AF did not change significantly in all samples, indicating that they nearly did not participate in the hydration reaction before 28 days. These results agree with previous works [26,33,34,35], in which the hydration of C_2_S was not observed even after 90 days of hydration due to its very low reactivity.

(3) $$3C_{4}A_{3}\bar{S}+2{\mathrm{CaCO}}_{3}+72H\;\to 2C_{3}A\cdot {\mathrm{CaCO}}_{3}\cdot H_{11}\left(\mathrm{Mc}\right)+C_{3}A\cdot 3C\bar{S}\cdot H_{32}\;\left(\mathrm{ettringite}\right)+6AH_{3}$$

**Figure 9:**
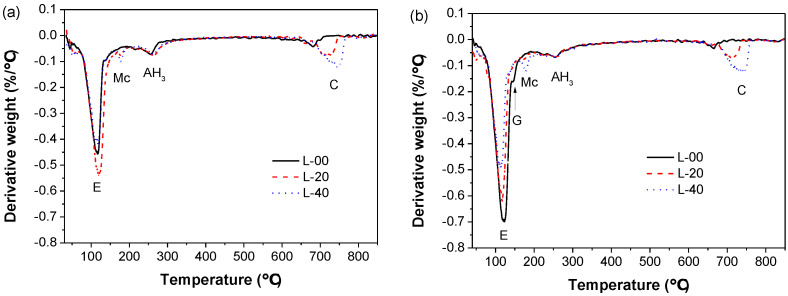
DTG curves of SCGM: (**a**) 6 h; (**b**) 28 days. (E: Ettringite; Mc: Mono‑carbonate; AH_3_: Aluminum hydroxide gel; C: Calcite; G: Gypsum).

The TGA test was also performed to reveal the phase assemblage of the hardened SCGM, and the DTG curves of L‑00, L‑20 and L‑40 cured for 6 h and 28 d are shown in Figure 9. The endothermic peak around 120 °C, 180 °C, 260 °C, and 720 °C was attributed to ettringite, Mc, aluminum hydroxide gel (AH_3_), and calcite, respectively [11,34,36,37]. Similar to the XRD and QXRD results, the primary hydrates phase assemblage was ettringite, Mc, and AH_3_. In the early ages, limestone powder would promote the formation of ettringite due to its nucleation effects. But the total amount of ettringite formed in the later ages was reduced as increasing the replacement rate of limestone powder, which was attributed to reduced anhydrite amount in the mixed slurry. Moreover, with the replacement rate of limestone powder increased from 20% to 40%, the weight loss due to the decomposition of Mc increased, indicating that more Mc was formed, which is in agreement with the XRD results.

#### 3.4.2. SEM Observation

**Figure 10:**
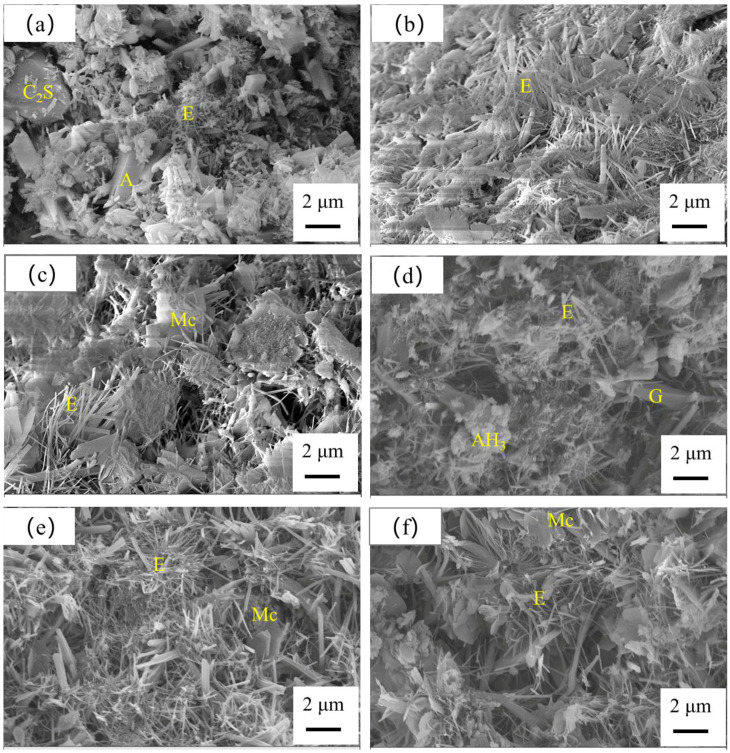
SEM images of the hardened SCGM cured for 6 h and 28 days: (**a**) L‑00 cured for 6 h; (**b**) L‑20 cured for 6 h; (**c**) L‑40 cured for 6 h; (**d**) L‑00 cured for 28 days; (**e**) L‑00 cured for 28 days; (**f**) L-00 cured for 28 days. (E: Ettringite; Mc: Mono-carbonate; AH_3_: Aluminum hydroxide gel; A: Anhydrite; G: Gypsum).

The SEM images of L‑00, L‑20 and L‑40 cured for 6 h and 28 days are shown in Figure 10. From Figure 10a, it was observed that lots of needle‑shaped ettringite were formed, and there were still un‑hydrated C_2_S and anhydrite. Further cured for 28 days, more ettringite was formed, and a denser microstructure was observed, as shown in Figure 10d. From Figure 10b,e mm more ettringite with a higher aspect ratio could be formed in the samples of L‑20 compared with L‑00. Flake Mc was also found, and the microstructure was denser. 

By comparison, for the sample of L‑40, ettringite and Mc were also formed, but the microstructure was looser compared with L‑00 and L‑40. It was reported that the formation of ettringite was responsible for the strength development of CSA cement [26,38,39]. Therefore, the increased ettringite amounts and densified microstructure contributed to the hardening strength of the L‑20 sample.

#### 3.4.3. Porosity Evolution

**Figure 11:**
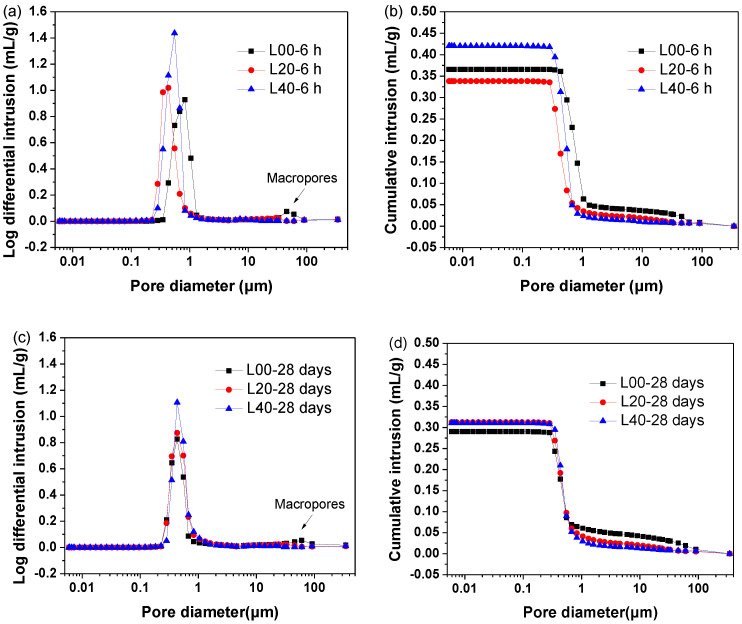
Pore structures of the hardened SCGM cured for 6 h and 28 days: (**a**) derivative curves of samples cured for 6 h; (**b**) cumulative curves of samples cured for 6 h; (**c**) derivative curves of samples cured for 28 days; (**d**) cumulative curves of samples cured for 28 days.

The porosity of L‑00, L-20 and L‑40 cured for 6 h and 28 days are shown in Figure 11, and the detailed porosity is tabulated in Table 5. For the SCGM cured for 6 h, it was clearly noted that with the replacement of anhydrite with limestone powder, the average pore diameter and total porosity of SCGM was reduced, especially for the sample of L‑20. The average pore diameter for the sample of L-00, L-20 and L-40 cured for 6 h was 0.77428 μm, 0.46227 μm and 0.51232 μm, respectively. The refined porosity was attributed to the formation of more ettringite in the presence of limestone powder due to its nucleation effects in the early age, as evidenced by XRD, TGA and SEM results. Further cured for 28 days, the average pore diameter and total porosity of SCGM could be further reduced.

The total porosity for the samples of L-00, L-20 and L-40 cured for 28 days was 30.29%, 29.64% and 27.23%, respectively. Also, it should be noticed that there were macropores in the L-00 samples cured for both 6 h and 28 days. However, in the presence of limestone powder, these macropores disappeared. This clearly confirmed the refinement of porosity by limestone powder.

**Figure 12:**
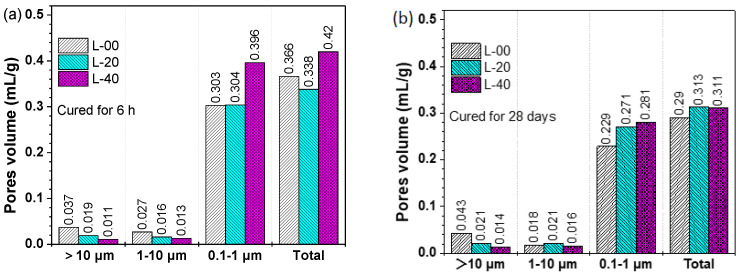
Effects of limestone replacement on intruded pores volume of hardened SCGM in terms of pores fractions: (**a**) SCGM cured for 6 h; (**b**) SCGM cured for 28 days.

More specifically, the intruded pore volume was plotted against three pore size regions as shown in Figure 12, in which the total volume of pores was labeled. For all samples, the volume fraction of pores between 0.1 μm and 1 μm was greater than 70% of the total pores volume. These pores were assumed to be the pores created by the intersection of ettringite crystals, as shown in Figure 10. And the pores smaller than 0.1 μm, that is, the gel pores, were not observed. This may because there were no C–S–H gels formed in this system. Consistent with the above results, in the presence of limestone powder, the macropores (d > 1 μm) were reduced in all L-20 and L-40 samples, indicating the physical filler effect of limestone powder.

## 4. Conclusions

In this work, the influence of the replacement rate of anhydrite by limestone powder on the hydration, hardening strength, expansion, and microstructure evolution of the SCGM was systematically investigated. The main findings can be drawn as follows:

(1) The replacement of anhydrite by limestone in slurry B would improve its flowability, shorten the setting time, and accelerate the hydration reaction of the mixed grouting material.
(2) Replacing anhydrite with less than 20% limestone, both the early and later compressive strengths were increased significantly compared with that of the L-00 sample. Further increasing the replacement rate to 40%, the later compressive strength was decreased, while the early compressive strength was still increased.
(3) The L-20 sample achieved the maximum compressive strength. Compared with L-00, the compressive strength for 6 h, 1 day, 3 days, 7 days, and 28 days was increased by 146.41%, 84.29%, 35.54%, 22.11%, and 22.33%, respectively.
(4) L-00 samples showed an expansion rate of 0.35% by 1 day, and the expansion rate of L-10 was similar to that of L-00. However, when the replacement rate of limestone powder was further increased, the expansion level was decreased.
(5) The primary hydrates phase assemblage of SCGM was ettringite, Mc and AH_3_. In the early ages, limestone powder would promote the formation of ettringite due to its nucleation effects. But the total amount of ettringite formed in the later ages was reduced as increasing the replacement rate of limestone powder.
(6) Enhancement of the hardening performance of SCGM by limestone powder was attributed to the increased ettringite amount, the densified microstructure, and the refined porosity.
(7) Moreover, the replacement rate of anhydrite by limestone powder may also affect the toughness or fracture behavior of the SCGM [40], and the related works will be conducted in the future.

## Figures and Tables

**Table 1:** Chemical composition of raw materials /wt.%.

Component	CaO	SiO_2_	Al_2_O_3_	Fe_2_O_3_	MgO	TiO_2_	SO_3_	Loss
CSA Clinker	41.53	6.36	38.27	1.27	1.15	1.77	8.88	0.17
Lime	64.72	3.46	0.67	0.48	2.1	—	—	27.58
Anhydrite	38.63	2.44	0.23	0.18	2.64	0.64	50.11	4.84
Limestone	54.45	1.12	0.47	0.09	0.54	0.02	0.15	43.12

**Table 2:** Mineral composition of CSA clinker /wt.%.

$C_{4}A_{3}\bar{S}$	β‑C_2_S	C_4_AF	f‑SO_3_	f-CaO	CaO·TiO_2_
74.54	18.25	3.86	0.81	2.02	3.01

**Table 3:** Mixture proportion of SCGM.

Mix No.	Slurry A		Slurry B			
	CSA Clinker	Water	Lime	Anhydrite	Limestone	Water
L‑00	500	600	100	400	0	600
L‑10	500	600	100	360	40	600
L‑20	500	600	100	320	80	600
L‑30	500	600	100	280	120	600
L‑40	500	600	100	240	160	600

**Table 4:** Setting time of the mixed slurry.

Mix No.	Initial Setting Time (min)		Final Setting Time (min)	
	Absolute	Relative	Absolute	Relative
L-00	5.0	100.0	7.5	100.0
L-10	4.5	90.0	6.0	80.0
L-20	2.7	54.0	5.5	73.3
L-30	2.5	50.0	5.0	66.7
L-40	1.4	28.0	4.8	64.0

**Table 5:** Summary of porosity and density results of hardened SCGM cured for 6 h and 28 days.

Mix No.	Porosity (%)	Average Pore Diameter (µm)	Total Intrusion Volume (mL/g)	Bulk Density (g/mL)
L-00-6h	31.71	0.77428	0.3659	0.8665
L-20-6h	29.78	0.46227	0.3384	0.8799
L-40-6h	34.79	0.51232	0.4203	0.8276
L-00-28d	30.29	0.52180	0.2904	1.0432
L-20-28d	29.64	0.49943	0.3127	0.9481
L-40-28d	27.23	0.50505	0.3107	0.8766
